# Exploring the Climatic Niche Evolution of the Genus *Falco* (Aves: Falconidae) in Europe

**DOI:** 10.3390/biology13020113

**Published:** 2024-02-11

**Authors:** Simona Mariana Popescu, Cristian Tigae, Aurelian Dobrițescu, Dragoș Mihail Ștefănescu

**Affiliations:** 1 Department of Biology and Environmental Engineering, University of Craiova, A.I. Cuza, 13, 200585 Craiova, Romania; simona.popescu@edu.ucv.ro; 2 Faculty of Science, University of Craiova, A.I. Cuza, 13, 200585 Craiova, Romania

**Keywords:** falcons, niche overlap, niche divergence, phylogenetic niche conservatism, convergent evolution, rate of climatic niche evolution

## Abstract

**Simple Summary:**

Falcons are keystone species that maintain ecosystem structures, functions, and services. With the help of phylogenetic comparative methods, climatic data, and species distribution modeling, we investigated how European falcon climatic niches have changed over evolutionary time. Convergent evolution and niche divergence have played an important role in the evolutionary history of these species, with speciation being influenced by climatic niche differentiation, more prevalent in the last 4 million years, with the main climatic niche shifts occurring between closely related falcon species.

**Abstract:**

By integrating species distribution modeling techniques, phylogenetic comparative methods, and climatic data, we analyzed how European falcon climatic niches have changed over evolutionary time in order to understand their tempo and mode of evolution and gain phylogenetic insights related to the ecological context of falcon evolution. For this purpose, we tested the relative contributions of niche conservatism, convergent evolution, and divergent evolution in the evolutionary history of this group of species in Europe. The occupation of climatic niche spaces by falcon species in Europe was not similar, considering that their climatic niche evolution was characterized by heterotachy, especially after ca. 4 Mya. Our results indicate that convergent evolution and niche divergence played an important role in the evolutionary history of these species, with no significant evidence of closely related species retaining their fundamental niche over time (phylogenetic niche conservatism). In most analyses, less closely related falcon species occupied similar climatic environments. We found that speciation in the European genus *Falco* was influenced by climatic niche differentiation, more prevalent in the last 4 million years, with the main climatic niche shifts occurring between closely related falcon species.

## 1. Introduction

The climatic niche of a species, as a central concept in ecology and evolutionary biology [1], is a key component of the overall niche space [2] and represents the set of environmental conditions associated with its occurrence [3,4] or the multidimensional hypervolume of climatic conditions in which a species exists [2]. The importance of the climatic niche derives primarily from the fact that climate is one of the main drivers of species distribution on a broad scale [5]. The environmental space available to a species is not fixed and changes over time [6], making the species respond to this change by evolving new physiological tolerances to resist this change, or by changing their distribution area or moving spatially, to maintain the relationship between their physiological functions and the particular climate that defines their niche [7,8]. The presence of niche conservatism, the tendency of a species to retain ancestral ecological characteristics [9], can limit the geographic range of the species due to its inability to adapt to new conditions [3], or to disperse [5,9], so the ancestral niche of a species determines the habitats and regions where the species could spread [10]. In addition, a high degree of climatic niche conservatism, with a slow evolution of the species’ climatic tolerance in the past, is associated with population demographic decline. This means that species with higher rates of past niche evolution could be more resilient to current climatic challenges [11,12], considering that species are not able to keep up with climate change as long as their rates of climatic niche evolution are much slower than the rate of future climate change [13,14,15].

While slow rates of climatic niche evolution limit species’ ability to adapt and persist through climatic perturbations [15], niche evolution enables adaptation to new climatic regimes that had previously limited species’ distributions and the expansion of niche breadth [10]. Niche divergence is a common process in nature, allowing species to adapt to environmental conditions that are different from those of their ancestors [2], and promotes ecological speciation through divergent natural selection (stemming from environments and resource competition) as species adapt to new environments [16,17].

As top predators, falcons (like other raptor bird species) are keystone species, maintaining ecosystem structures, functions, and services [18]. The rapid diversification of falcons in the Early–Middle Miocene [19], with a species richness of the genus *Falco* 8 to 9 times higher than that of any other genus of birds [20], offers phylogenetic insights regarding the factors that have driven its evolution [20], considering that understanding how lineages have diversified over time is evolutionary biology’s central question [19]. Falcons (more precisely, falcons and caracaras) belong to the order Falconiformes, with one family, the family Falconidae, comprising 11 genera with 65 recognized species [21] spread throughout the world (except the Antarctic, very remote oceanic islands, and the high arctic) in very diverse habitats, from forests to steppes and arctic zones, and also human habitats, like city centers [22]. Three subfamilies are recognized within the Falconidae family: Herpetotherinae (comprising two genera and eight species); Polyborinae (comprising six genera and eleven species); and Falconinae (comprising three genera and forty-six species) [19,21].

In Europe, there are ten falcon species (all belonging to the genus *Falco*) recognized as breeding species [23]: Merlin (*Falco columbarius*), Common Kestrel (*Falco tinnunculus*), Lesser Kestrel (*Falco naumanni*), Red-footed Falcon (*Falco vespertinus*), Eleonora’s Falcon (*Falco eleonorae*), Eurasian Hobby (*Falco subbuteo*), Peregrine Falcon (*Falco peregrinus*), Lanner Falcon (*Falco biarmicus*), Saker Falcon (*Falco cherrug*), and Gyrfalcon (*Falco rusticolus*). The last three species are members of the hierofalcons, a group of closely related species, which are not clearly genetically differentiated, with hybrid zones between populations [24]. We considered the Barbary Falcon (*Falco pelegrinoides*) as a subspecies of the Peregrine Falcon (*Falco peregrinus*), based on slightly distinct genetic differences, in [25], although some authors have suggested that this subspecies should be recognized as a separate species [19] based on molecular, morphological, and behavioral data. Also, we treated Merlin as *Falco columbarius* in [21] and not as *Falco aesalon* [19,20]. Therefore, the focus group of the present study was formed of all ten species of the genus *Falco* present in Europe as regular breeding species.

Despite the fact that representatives of this genus in Europe form a polyphyletic group, with most species of the genus not being present on this continent, for simplicity, we considered this group of species as a clade, from a geographical perspective, considering that our research focused on assessing the evolutionary history of this group at the regional level, with this clade containing all of the known descendants of a single common falcon ancestor that populate Europe’s habitats.

Given that the species of the genus *Falco* in Europe occupy a great variety of climatic zones, from arctic to Mediterranean subtropical ones, we aimed to increase the knowledge about the tempo and the mode of falcon climatic niche evolution in Europe by combining climatic data (conducted by ordination and potential distribution methods) with phylogenetic comparative methods. Our main objectives were to: (1) identify the current position of the falcon species within the multidimensional climatic niche space, highlighting their degree of specialization and overlapping, in order to test for niche conservatism and niche divergence; (2) estimate the ancestral climatic tolerances, by reconstructing their niche in both geographic and environmental spaces; (3) compare different macroevolutionary models to test for various falcon climatic niche evolution scenarios; and (4) estimate their rates of climatic niche evolution.

## 2. Materials and Methods

### 2.1. Species Occurrence Points, Climatic Data, and Species Distribution Modeling

The presence points for the ten species of the genus *Falco* present in Europe were obtained through the Global Biodiversity Information Facility (GBIF) platform (https://www.gbif.org/ accessed on 1 April 2022), accessing databases from the EBCC Atlas of European breeding birds and the eBird Observation Dataset. In order to identify records that were imprecise (e.g., lack of geographical information, points of presence outside of the knowing distribution area of the species, points in oceans, etc.) and to reduce sampling biases in subsequent analyses, we used the *CoordinateCleaner* library [26] in R ([27], https://cran.r-project.org/ accessed on 2 January 2022) to clean occurrence records retrieved from GBIF. After cleaning, we retained a total of 12,723 falcon occurrence points for further analyses.

Temperature and precipitation information used to infer the present climatic niches of falcon species was acquired from the WorldClim database (https://www.worldclim.org, accessed on 13 June 2021) as 19 bioclimatic variables (Supplementary Table S1) at a 2.5 min (≈5 km) spatial resolution. We searched for multicollinearity in our data with the *usdm* library in R [28], using a correlation coefficient threshold of 0.7. After excluding collinear variables, we retained seven climatic variables for modeling the climatic evolution of falcons in Europe: annual mean temperature (Bio1), annual mean diurnal range (Bio2), annual temperature range (Bio7), mean temperature of wettest quarter (Bio8), precipitation seasonality (Bio15), precipitation of warmest quarter (Bio18), and precipitation of coldest quarter (Bio19).

The potential distribution of falcon species in Europe in relation to climatic variables was modeled using the Bioclim algorithm in the *dismo* library in R [29]. Despite its simplicity, by using this algorithm, it is highly probable that areas identified as suitable for a species are correctly identified [30]. Model fit was assessed with the AUC (Area Under Curve) metric as implemented in the *dismo* library.

### 2.2. Niche Overlap and Niche Breadth Estimation

Estimation of falcon species niche overlap was conducted in both geographic and environmental spaces [31,32], considering that the limitation of many comparative niche studies come from the fact that niche overlap is quantified in geographic space and not in environmental space also [31]. Our geographical niche overlap method, as implemented in the *dismo* library (*nicheOverlap* function), is based on prediction (geographical projections) of species niches as inferred by SDMs. Using this function, we calculated Schoener’s D statistic [29,33] for each falcon species pair, an index that takes values from 0 (no overlap) to 1 (complete overlap). The PCA-env method used by Broennimann et al. [34] was used for niche overlap quantification in environmental space, as implemented in the *ecospat* library in R [35], by calculation of Schoener’s D statistic from the first and second principal components, derived from principal component analysis (PCA). Niche breadth for each species was determined in the *ENMTools* version 1 library in R [36] with the *raster.breadth* function and the B2 Levins metric [37]. This metric’s values range from 0 to 1 and represent the range of conditions tolerated by a species. Furthermore, we ran an equivalency and similarity test [36,38] for each falcon species pair (based on species occurrence density grids), as implemented in the *ecospat* library, to test for niche divergence and niche conservatism. We classified the overlap values according to Rödder and Engler [39] as follows: no or very limited overlap (0–0.2), low overlap (0.2–0.4), moderate overlap (0.4–0.6), high overlap (0.6–0.8), and very high overlap (0.8–1.0).

### 2.3. Phylogeny and Diversification

We constructed a maximum clade credibility tree (MCCT) using median node heights in TreeAnnonator software v2.6.6 (BEAST package, [40]) from a subset of 1000 fully resolved Markov chain Monte Carlo (MCMC) phylogenies for the ten *Falco* species present in Europe. They were downloaded from the BirdTree project (https://birdtree.org/ accessed on 12 March 2022), which represents the first set of complete phylogenies of extant bird species based on a Bayesian framework [41]. The MCCT was used for the subsequent phylogenetic analyses. Mean node ages and the uncertainty of inferred divergence times expressed by 95% highest posterior densities (HPD) were extracted from the MCCT also. The HPD is the shortest interval that contains 95% of the posterior probability density.

In order to calculate temporal falcon diversification in Europe based on the MCCT, we created a lineage-through-time (LTT) plot, as implemented in the *phytools* version 1.0-3 library in R [42], by counting lineage numbers as they accumulate from the root of the tree up to the present day. Under a pure-birth model (no extinction and constant diversification rate through time), the LTT follows a straight line on the logarithmic scale (exponential increase). To take incomplete sampling into consideration, we conducted a Monte Carlo constant rates (MCCR) test [42,43] implemented in the *phytools* library, calculating the gamma statistic both for MCCT and for the 1000 trees set sampled from the BirdTree search. In addition, we compared the fit of seven alternative diversification models based on Condamine et al. [44] and Revell and Harmon scripts [45], *DDD* [46], *diversitree* [47], and *phytools* [42] libraries. Among the seven models, two models assumed a constant rate of diversification (a pure-birth model—Yule, with no extinction, and a constant-rate birth–death model—crBD, with extinction but with constant rates for extinction and speciation), three of them assumed variable speciation and/or extinction (variable speciation model—vS, variable extinction model—vE, and variable speciation and extinction model—vSE), and the last two models assumed a diversity-dependent diversification (density-dependent linear model—DDL + E, and density-dependent exponential model—DDX + E), taking into account the influence of the changes of species accumulation over time on the rate of diversification. The best-fit model of diversification was selected based on the AIC approach. In addition, a likelihood ratio test was conducted for the two best models.

### 2.4. Niche Evolution Analyses

We used phylogenetic principal components analysis, taking into account the nonindependence of species when computing the correlations between climatic variables, to explore the distribution of the genus *Falco* in the multivariate climatic niche space using the *phyl.pca* function in the *phytools* library. Subsequently, we quantified the phylogenetic signal for each climate variable to identify the extent to which closely related species share each climatic niche dimension, considering that phylogenetic signal is the tendency of related species to be more similar than distantly related species [48], with a strong phylogenetic signal suggesting that closely related species share similar traits/traits values for continuous traits, while more distantly related species are less similar to each other [49]. Phylogenetic signal was tested for each climatic variable with the *phyloSignal* function in the *phylosignal* library in R [50], with four different models: Blomberg’s K, Pagel’s Lambda, Moran’s I, and Abouheif’s Cmean. Estimation of *p*-values was performed through randomization (999 repetitions).

To analyze the climatic niche evolution of the genus *Falco* in Europe, we generated predicted niche occupancy (PNO) profiles as implemented in the *phyloclim* library in R [51], by integrating the Bioclim probability distribution of each species and for each climatic variable in the form of a unit area histogram of suitability, representing species’ tolerance in accordance with respective climatic variables [52]. After that, the ancestral climatic tolerance of falcon species in Europe was reconstructed for each climatic variable, in both geographic and environmental spaces [32], by combining the phylogenetic data (MCCT) with species’ climatic information: predicted niche occupancy profiles (PNOs), for geographic space; and the median of climatic values extracted from species occurrence points, for environmental space (Supplementary Table S2). The ancestral reconstruction of climatic niche evolution in environmental space was conducted using the *phytools* function *fastAnc*, followed by plotting the results on a phenogram [42]. Weighted means of the PNOs for each falcon species and climatic variable were used for all subsequent analyses of climatic niche evolution (Supplementary Table S3).

We used the *mvMORPH* library in R [53] to assess the fit of five alternative evolutionary models for each of the seven bioclimatic variables and to uncover the mode of falcon species’ climatic evolution in Europe. Two of these five models require a prior establishment of regimes, based here on the latitudinal position on which the geographical range of falcons is centered, according to the Finlayson classification [54]: *general* (multilatitude species: *F. peregrinus*, *F. tinnunculus*, and *F. subbuteo*), *north* (arctic, boreal and temperate species: *F. rusticolus*, *F. columbarius*, and *F. vespertimus*), and *south* (mid-latitude and subtropical species: *F. cherrug*, *F. naumanni*, *F. biarmicus*, and *F. eleonorae*). This division of species within these three regimes allows us to test if there is a significant climatic convergence of falcons according to latitude (distinct climatic optima for the three regimes). The five models are as follows: (1) a single-rate Brownian model (BM1) according to which the traits evolve as a random walk over time and a constant evolutionary rate [55]; (2) a multi-rate Brownian model (BMM), with different rates of niche evolution for each regime [56,57]; (3) a constrained (single-optimum) Ornstein–Uhlenbeck model of evolution (OU1), involving evolutionary change toward a single optimum [58,59]; (4) a multiple-optima Ornstein–Uhlenbeck model of evolution (OUM), which allows for different optima for each regime [60]; and (5) an early-burst (EB) model according to which traits evolve rapidly, soon after the beginning of lineage diversification (adaptive radiation), followed by a slowdown in the rate of niche evolution [61]. To account for uncertainty, the five evolutionary models were fitted over 1000 stochastically mapped trees, for each climatic trait, using Bayesian stochastic character mapping [62], as implemented in the *phytools* library. First, 100 trees were randomly selected from the posterior distribution (1000 fully resolved MCMC phylogenies) and then for each of them, 10 stochastically mapped trees were generated. Next, parameter estimates and AIC_c_ values were calculated for each simulation and then averaged over all 1000 simulations for each model and variable. Thus, AIC_c_ wights were calculated from mean AIC_c_ values, for each climatic variable, and the model with the highest AIC_c_ weight was selected as the best model [63]. We used ΔAIC to rank models [64,65], where, as a rule of thumb, models having ΔAIC < 2 are more or less equivalent, models with ΔAIC within 4–7 are distinguishable, and models with ΔAIC > 10 are different. Moreover, we followed the methods of Martínez-Méndez et al. [65] to classify the models as follows: equivalent models (ΔAIC < 2), more or less distinguishable models (ΔAIC ≥ 2 and < 7), distinguishable models (ΔAIC ≥ 7 and <10), and different models (ΔAIC ≥ 10).

To test the effect of latitude on the mean climatic tolerances of falcons (weighted means of PNOs), for all climatic variables, and the relationship between range size and climatic niche breadth for falcon species, we used Phylogenetic Generalized Least Squares (PGLS). Furthermore, to check for Rapoport’s rule [66], we plotted the latitude against species’ range size using *ggplot2* [67] and *letsR* [68] libraries. To test for the convergent evolution of falcon species on the climatic niche axis, the Wheatsheaf index using the *windex* library [69] was employed also. The Wheatsheaf index generates Euclidean distances between species from climatic axes and penalizes them by species relatedness before proceeding to convergences.

In order to estimate and detect changes in the rate of climatic niche evolution, we applied BAMM version 2.5.0 (Bayesian Analysis of Macroevolutionary Mixtures), a C++ program [70,71]. This model was used both for estimation of speciation–extinction rates (diversification) and climatic evolutionary rates across the MCCT.

### 2.5. Age Range Correlation

To infer the prevalence of allopatric versus sympatric speciation, we used the age range correlation (ARC) analysis proposed by Fitzpatrick and Turelli [72] and implemented it in the *phyloclim* library with the *age.range.correlation* function, to examine how average niche overlap is regressed against node ages. Niche overlap based on Schoener’s D statistic was calculated from species’ Bioclim probability surfaces in ASCII format. If allopatric speciation is prevalent, the range overlap at each node decreases in time, so recently diverged nodes are less similar than more ancestral nodes [73]. Furthermore, we conducted a linear regression analysis to explore the relationship between the niche overlap values at internal nodes of the MCCT and BAMM rates of climatic niche evolution, for each variable, in order to quantify the importance of the rate of climatic evolution in species divergence through time. BAMM rates were estimated for each node of the MCCT with the function *getCladeRates* in the BAMMtools library.

## 3. Results

### 3.1. Environmental Niche Modeling: Niche Analysis

The SDMs obtained by the Bioclim algorithm showed very good fits (according to the AUC criterion), with AUC values ranging from 0.835 in *F*. *tinnunculus* to 0.996 in *F*. *cherrug*. Species with the largest predicted ranges presented the lowest AUC values, as in the case of *F*. *tinnunculus* (0.835), *F*. *peregrinus* (0.852), and *F*. *subbuteo* (0.867), while the other species, except *F*. *vespertinus* (0.882), showed AUC values > 0.9 (*F. columbarius*: 0.903; *F. biarmicus*: 0.905; *Falco eleonorae*: 0.925; *F. rusticolus*: 0.962; *F. naumanni*: 0.968; *F*. *cherrug*: 0.996). Further, the predicted distributions obtained were consistent with known falcon distribution ranges in Europe (Supplementary Figure S1).

The largest climatic niches among falcon species that occurred in Europe were found for *F. tinnunculus* (0.578), *F. peregrinus* (0.519), and *F. subbuteo* (0.500), these three species being the most widespread on the European continent. The most specialized species in terms of climate are *F. naumanni* (0.110), *F. biarmicus* (0.130), *F. cherrug* (0.159), and *F. eleonorae* (0.180), which are subtropical species (excepting for *F. cherrug*), with their geographic range centered on the 30–40° N latitudinal band. *F. vespertinus* (0.368), *F. columbarius* (0.248), and *F. rusticolus* (0.204) occupy intermediate positions regarding climatic specialization.

The niche overlap varied not only among falcon species but also when niche overlap was measured in geographic space (g-space) or environmental space (e-space). In general, niche overlap among falcon species was very limited to low, according to the Rödder and Engler classification [39]. In the g-space, niche overlap values ranged from 0.030 to 0.881 (Supplementary Table S4), with an average value of 0.344 ± 0.230 (mean ± SD), and in the e-space, niche overlap values ranged from 0.025 to 0.846 (Supplementary Table S5), with an average value of 0.308 ± 0.221. In both cases, as expected, high (0.6–0.8) and very high niche overlap values (0.8–1.0) were recorded between the three generalist species, *F. tinnunculus*, *F. subbuteo*, and *F. peregrinus* (Supplementary Tables S4 and S5), with one exception of a high niche overlap value for *F. biarmicus*—*F. eleonorae* (0.683 in e-space, and 0.623 in g-space), in regard to the other falcon species apart from the generalist ones. Moreover, observed niche overlap values between species belonging to the same subclade ranged from very limited overlap for hierofalcons (0.090 ± 0.023 in e-space; 0.062 ± 0.034 in g-space) to low overlap for *F. subbuteo*—*F. eleonorae* (0.395 in e-space; 0.396 in g-space) and moderate overlap for *F. tinnunculus*—*F. naumanni* (0.428 in e-space; 0.316 in g-space).

A PCA randomization test for niche equivalency showed that, out of 45 niche overlap pairwise falcon species comparisons, 7/45 (15.6%) had equivalent niches, revealing significantly greater niche overlap than the null expectation, and 25/45 (55.6%) exhibited niche divergence, with significantly lower niche overlap than the expectation (Supplementary Table S5). The closest relative species pairs *F*. *tinnunculus*/*naumanni* and *F*. *subbuteo*/*eleonorae* are among those species pairs that have identical niches, suggesting a pattern of niche conservatism (Supplementary Table S5). In addition, the niche similarity randomization test could not identify any kind of niche divergence between species pairs based on available background environment. Instead, a similarity test revealed substantial evidence for niche conservatism, with 18/45 (40%) of comparisons indicating significant departure from the null expectation (greater niche overlap) in at least one direction (Supplementary Table S5), two of which were observed for the same two pairs of closest relative species: *F*. *tinnunculus*/*naumanni* and *F*. *subbuteo*/*eleonorae*.

### 3.2. Phylogenetic Relationships and Divergence Time Estimates

**Figure 1:**
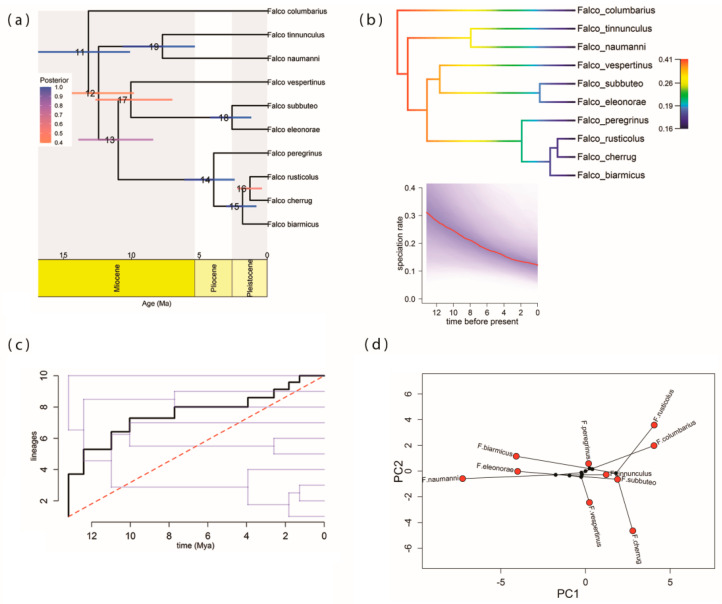
(**a**) Maximum clade credibility tree (MCCT) of genus *Falco* in Europe (see text for further information). Bars correspond to the 95% highest posterior density (HPD) confidence intervals of the node age, colored by the posterior probability of the clade. Each node of the MCCT is numbered. Ma: megaannum. (**b**) Rates of falcon species diversification (phylorate plot) inferred with BAMM, with branches colored according to the net diversification rate. Warmer colors represent faster rates. Inset plot shows speciation rates through time estimated with BAMM. Shaded area depicts 95% confidence interval. Time in millions of years. (**c**) Lineage-through-time plot (LTT) showing falcon species accumulation in time. The dotted red line represents the expected number of lineages under a pure-birth process. Mya: million years ago. (**d**) Phylogenetic PCA for multivariate climatic niche space, with PC1 and PC2 loadings plotted on x and y axes. Black lines indicate the phylogenetic relationships between species.

The coalescence time of the genus *Falco* in Europe, based on MCCT, was estimated to be ca. 13.60 Mya (95% HPD: 10.08–16.85 Mya) (Figure 1a) in the Middle Miocene, a time when *F. columbarius* split from the rest of the falcons (Figure 1a), forming a separate subclade. The divergence of the *F. tinnunculus*/*naumanni* subclade was dated in the Middle Miocene ca. 12.41 Mya (95% HPD: 9.80–15.53 Mya), a falcon species pair which subsequently split ca. 7.71 Mya (95% HPD: 5.32–10.63 Mya) in the Late Miocene (Figure 1a). The *F. vespertinus*/*subbuteo*/*eleonorae* subclade diversified in the Late Miocene, ca. 10.98 Mya (95% HPD: 8.38–13.90 Mya), and has subsequently diverged ca. 10.03 Mya (95% HPD: 6.97–12.64 Mya) and ca. 2.58 Mya (95% HPD: 1.17–4.20 Mya), between the Late Miocene and the Early Pleistocene. The divergence from the most recent common ancestor (MRCA) of the hierofalcons and *F. peregrinus* subclade was estimated ca. 3.93 Mya (95% HPD: 2.39–6.09 Mya), in the Early Pliocene, followed by two subsequent hierofalcon diversification points ca. 1.81 Mya (95% HPD: 0.78–3.02 Mya) and ca. 1.27 Mya (95% HPD: 0.37–2.19 Mya) in the Early Pleistocene (Figure 1a). *F. tinnunculus*/*F. naumanni* and *F. subbuteo*/*F. eleonorae* are not sister species, but at the European continental scale, these two falcon pairs are the closest living relatives, as a consequence of taxon sampling. Thus, in Europe, three falcon species pairs have an exclusive common ancestor, but only *F. cherrug*/*F. rusticolus* is a true sister pair.

### 3.3. Pattern of Lineage Diversification

BAMM analysis found no shifts in rates of falcon diversification. The best supported shift configuration with posterior probability = 1 is provided as a phylorate plot in Figure 1b, indicating a reduction in estimates of instantaneous net falcon diversification rate over time, due to a strong reduction in speciation rates through time within the falcon clade, as estimated by pulled speciation rates (Figure 1b-inset). The lineage-through-time plot (LTT) on falcon phylogeny indicated the presence of a slightly early burst of diversification, followed by a constant rate of diversification in the last ca. 4 Mya (Figure 1c), with the gamma statistic calculated for both the MCCT (γ = −0.780, *p* = 0.453) and for 1000 trees randomly sampled from the posterior distribution of the fully resolved Markov chain Monte Carlo (MCMC) search (γ = −0.811, *p* = 0.432), suggesting that lineage accumulation over time in European falcons does not differ from a pattern expected under a model of constant-rate diversification. Complementarily, model selection analyses of lineage diversification identified the pure-birth model (Yule model) as the best fitting approximation of falcon diversification in Europe, based on AIC (Table 1), although the likelihood ratio test between this model and the second ranked model (constant-rate birth–death model) was non-significant ($\chi_{1}^{2}\;$= 0.001; *p* = 0.966).

### 3.4. Climatic Niche Evolution

Phylogenetic principal component analysis showed that the first two principal component axes accounted for more than 90% of the total climatic variation, with the first PC axis accountable for 51.07% of the total variance and the second PC axis accountable for 44.03%. Both main PC axes captured, primarily, a gradient in precipitation (Supplementary Table S6), with the most pronounced positive loading for precipitation of the warmest quarter (Bio18) and the highest negative loadings for precipitation seasonality (Bio15) and annual mean temperature (Bio1) for the first PC axis and the highest positive loading for precipitation of the coldest quarter (Bio19) and the highest negative loadings for mean temperature of the wettest quarter (Bio8) and annual temperature range (Bio7) for the second PC axis. There is an obvious clear separation of falcon species in the European climatic niche space formed by the first two PC axes (Figure 1d), with *F. peregrinus*, *F. tinnunculus*, and *F. subbuteo* occupying the central part of this space, indicating a high climatic tolerance of these species, with their climatic positions close to the average climatic tolerance values of European falcons (Supplementary Tables S2 and S3), and two areas of climate specialization within the phylospace: one grouping of *F. naumanni*, *F. eleonorae* and *F. biarmicus*, which prefer habitats with lower rainfall in the warmest quarter of the year, high seasonality of rainfall and higher temperatures; and another grouping *F. rusticolus* and *F. columbarius* and, to a lesser extent, *F. cherrug*, occupying a climatic niche space dominated by high amounts of precipitation during the warmest period of the year. In addition, these last three falcon species are quite well differentiated on the second PC axis, with *F. cherrug* positioned in a climatic space dominated by high temperatures in the wettest season and high values for ranges of extreme temperature conditions and *F. rusticolus* tolerating areas dominated by high rainfall during the coldest period of the year. *F. columbarius* occupies an intermediate position between these two species, regarding the last variable. *F. vespertinus* is placed between the two areas of climatic specialization of the niche space, closer to the group of generalist species (Figure 1d).

We did not detect a statistically significant phylogenetic signal for any metric or any bioclimatic variables retained for analysis, so variables were less similar than expected under a Brownian motion model (Supplementary Table S7).

**Figure 2:**
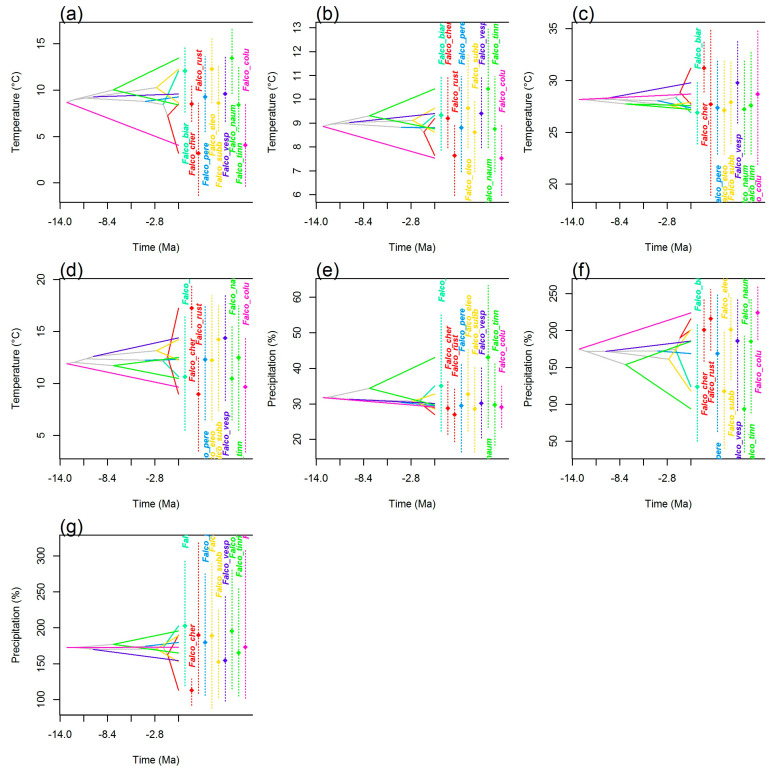
Inferred history of the evolution of climatic tolerance in European falcon clade based on MCCT. Interior nodes indicate the mean of climatic tolerance inferred for the most recent common ancestor of the extant falcon species. Each species is represented by a color that is preserved from one variable to another. For each species, the point indicates the mean climatic tolerance and the vertical dashed line (of the same color) indicates the 80% central density of climatic tolerance. Species names consist of the first letter of the genus and the first three letters of the species name. (**a**) annual mean temperature; (**b**) annual mean diurnal range; (**c**) annual temperature range; (**d**) mean temperature of wettest quarter; (**e**) precipitation seasonality; (**f**) precipitation of warmest quarter; (**g**) precipitation of coldest quarter.

**Figure 3:**
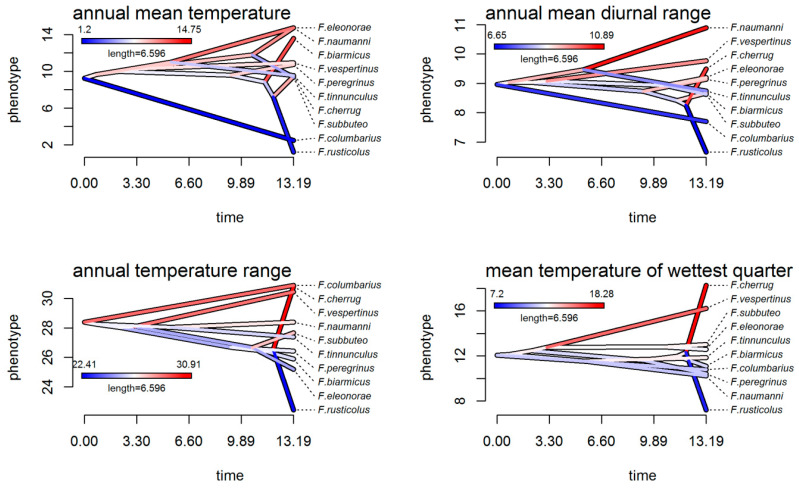
Ancestral climatic niche reconstruction in falcon clade across the MCCT for thermal niche axes in environmental space. Time in millions of years.

**Figure 4:**
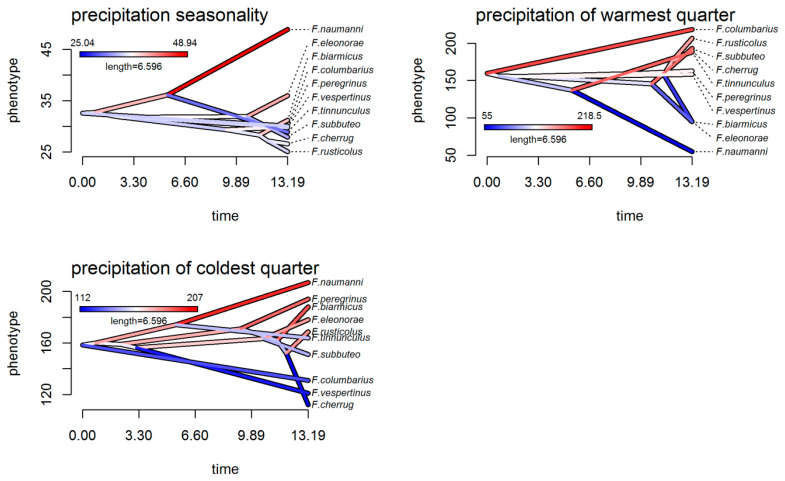
Ancestral climatic niche reconstruction in falcon clade across the MCCT for precipitation niche axes in environmental space. Time in millions of years.

Analyses of the climatic tolerance evolution of falcons, both in the g-space (Figure 2) and the e-space (Figure 3 and Figure 4), were in complete agreement and showed both the divergent and convergent evolution of these species, indicating that there has been a considerable evolution of climatic tolerance, with different patterns depending on the variable considered. In the climatic history of falcons in Europe, there were instances of species diverging from members of their own subclade and converging to a mean climatic tolerance, more similar to that of more distantly related species. For example, an extreme divergent evolution was highlighted in the *F. cherrug/rusticolus* sister pair considering mean temperature of the wettest quarter (Bio8), with higher mean thermic tolerance for *F. cherrug* (Figure 2 and Figure 3). Thus, for annual temperature range (Bio7) and mean temperature of the wettest quarter (Bio8) and to some extent for precipitation seasonality (Bio15), the falcon species showed a more convergent evolution.

**Figure 5:**
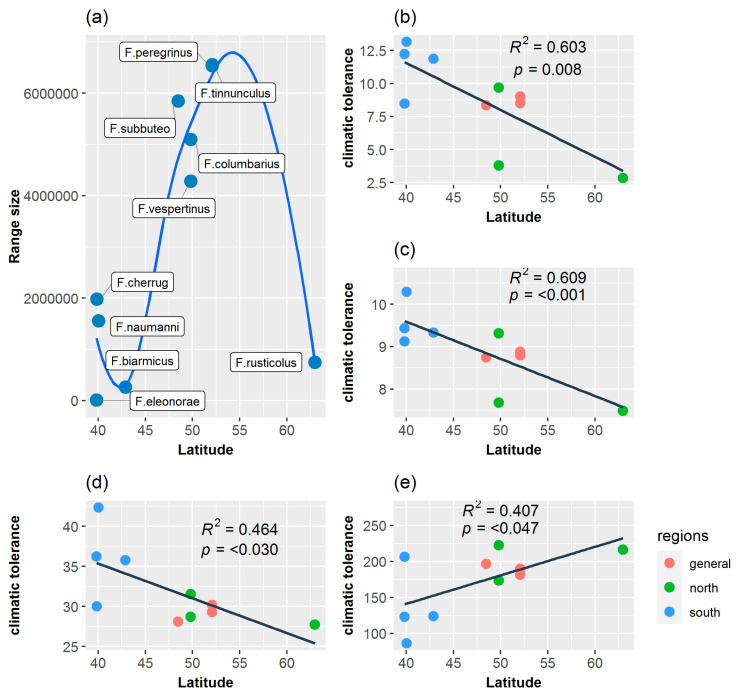
Relationships between latitude and falcon species range size (**a**) between latitude and mean climatic tolerance (weighted mean of the PNOs) for annual mean temperature (**b**), annual mean diurnal range (**c**), precipitation seasonality (**d**), and precipitation of warmest quarter (**e**). Colored points represent the falcon species categories based on latitudinal position on which their geographical range is centered (*general*: multilatitude species; *north*: arctic, boreal, and temperate species; *south*: mid-latitude and subtropical species). The grouping of the ten falcon species in the three categories is indicated in the methodology. Regression lines are produced using Phylogenetic Generalized Least Squares (PGLS).

mvMORPH analyses indicated the OU1 model as the best supported model of trait evolution for all seven bioclimatic variables, based on AICc (Table 2), although OU1 and BM1 are equivalent (ΔAIC < 2) or more or less distinguishable (ΔAIC ≥ 2 and <7) models (Table 2). Anyhow, the selection of the OU1 model over the BM1 model suggests that there is a possible stabilizing selection toward one climatic adaptive optimum. Moreover, although statistical analyses suggest that the latitudinal distribution of falcon species in Europe has no significant influence on their climatic tolerances (no statistical support for an OUM model of climatic evolution, Table 2), PGLS results (Figure 5) revealed a significant effect of latitude on falcon mean climatic tolerances (weighted means of PNO) for annual mean temperature (Bio1) (*F*_1,8_ = 12.160, *p* = 0.008), annual mean diurnal range (Bio2) (*F*_1,8_ = 50.180, *p* < 0.001), precipitation seasonality (Bio15) (*F*_1,8_ = 6.927, *p* = 0.030)*,* and precipitation of the warmest quarter (Bio18) (*F*_1,8_ = 12.16, *p* = 0.047), supporting the idea of an evolutionary model with more than one adaptive peak. Therefore, falcon species with subtropical and mid-latitude distributions have the highest values of mean climatic tolerance (highest weighted means of the predicted niche occupancy) for annual mean temperature (Bio1), annual mean diurnal range (Bio2), and precipitation seasonality (Bio15) and the lowest values for precipitation of the warmest quarter (Bio18), except for *F. cherrug* for the last variable. Moreover, there is a significant positive relationship between range size and climatic niche breadth of falcon species (PGLS: *F*_1,8_ = 38.16, *p* < 0.001, Supplementary Figure S2), indicating low-latitude falcon species having restricted geographical range and the highest degree of specialization, a consequence of the relationship between range size and latitude (Rapoport’s rule), (Figure 5a).

The Wheatsheaf index confirmed the convergence of falcon species with subtropical and mid-latitude distributions (except *F. cherrug*) in terms of annual mean temperature (Bio1) and precipitation of the warmest quarter (Bio18), where it was significantly different from that expected under a random distribution, suggesting a high strength of selection on these traits (*w* = 1.276, *p* = 0.032, 95% CI = 1.007–168.899). An obvious evolutionary convergence exists also between those species with arctic and boreal distributions (*F. rusticolus* and *F. columbarius*). This convergence was supported by the convergent strength test (Wheatsheaf index) for annual mean temperature (Bio1), as a consequence of the adaptation of *F. rusticolus* and *F. columbarius* to rather similar environments (*w* = 1.047, *p* < 0.001, 95% CI = 0.948–∞), both species showing the lowest values for weighted means of the predicted niche occupancy and medians of values for this climatic variable (Supplementary Tables S2 and S3).

**Figure 6:**
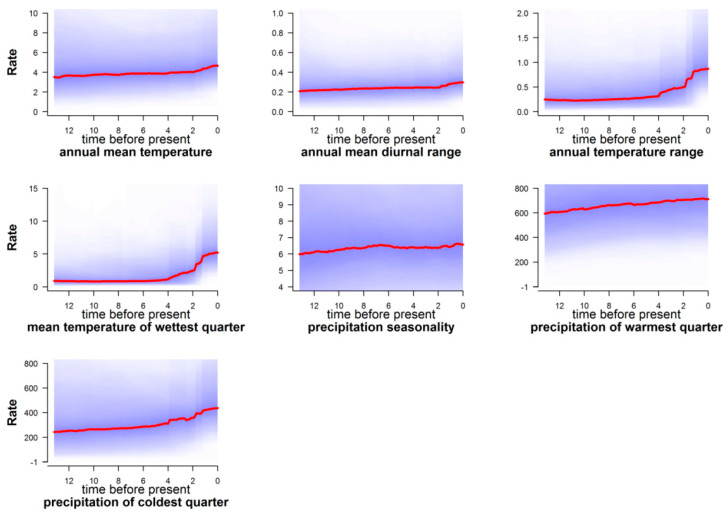
BAMM rate-through-time plots for rate of climatic evolution (with 95% CI represented by shaded area around red lines) in European falcon clade. Time in millions of years.

According to BAMM rate-through-time plots for climatic variables, there was an overall increase regarding the process of climatic trait diversification, from a subtle increase (Bio1, Bio2, Bio15, and Bio18) to a more pronounced one (Bio7, Bio8, and Bio19), that began ca. 4 Mya (Figure 6).

BAMM analysis indicated a heterogeneity of instantaneous rates of continuous climatic evolution of the falcon clade for Bio1, Bio2, Bio7, Bio8, and Bio19, mainly at node 14 (the estimated time of split between *F*. *peregrinus* and hierofalcons), node 15 (the estimated time of origin and diversification of the hierofalcons, *F*. *rusticolus*/*cherrug*/*biarmicus* subclade), and node 16 (the estimated time of origin of *F*. *rusticolus*/*cherrug* sister species) of the MCCT (Supplementary Figure S3), with no internal rate shifts, except for mean temperature of the wettest quarter (Bio8), in which case, the most frequently supported model (*f* = 0.37) included a single rate shift at node 15 (Supplementary Figure S3d). In all these cases, regardless of the presence of rate shifts or not, the BAMM model highlighted that the mean rates were higher for those species originated from nodes 14, 15, and 16 than those of the neighboring subclades, supporting an increase in climatic evolution rates for these variables.

### 3.5. Age Range Correlation

**Figure 7:**
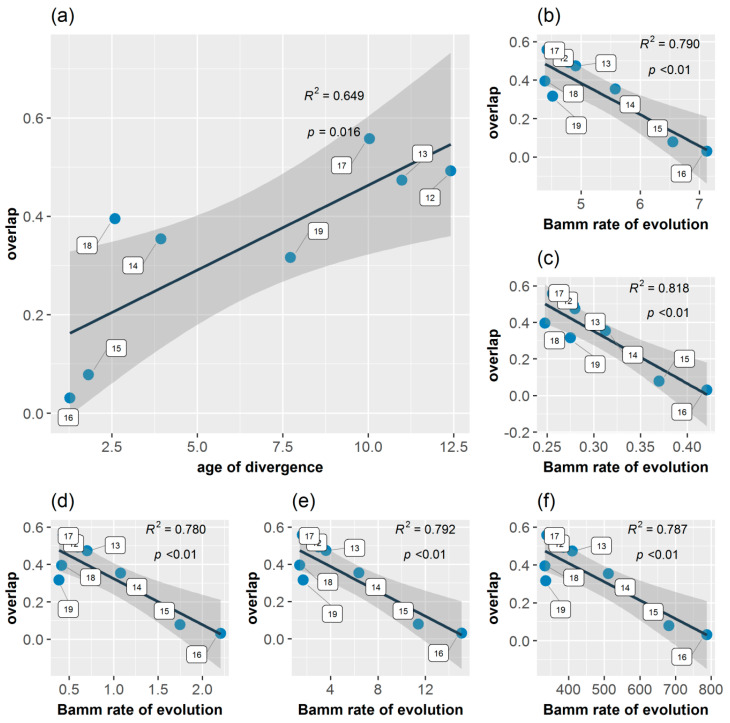
Scatterplots showing the relationships between the divergence time and the degree of range overlap (age range correlation) among closest relative species of falcons (**a**) and between the degree of range overlap and BAMM rate of climatic niche evolution for annual mean temperature (**b**), annual mean diurnal range (**c**), annual temperature range (**d**), mean temperature of the wettest quarter (**e**), and precipitation of the coldest quarter (**f**). Blue filled circle are nodes of the MCCT, indicated by the corresponding numbers (according to Figure 1a). The *R^2^* and *p*-values are listed for each relationship. Shaded grey areas around each regression line indicate 95% confidence intervals. Node 17 overlaps node 12.

Age range correlation analysis indicated that there was a non-significant positive correlation between divergence time and niche overlap at internal nodes (*R*^2^ = 0.308, *F*_1,7_ = 3.120, *p* = 0.121). The relationship became significant (*R*^2^ = 0.649, *F*_1,6_ = 11.120, *p* = 0.016) and more evident when the initial node (node 11) was removed (outlier), indicating recently diverged nodes being less similar than older ones (accumulation of niche differences with time), suggesting that climatic niche differentiation could have played a role in falcon diversification in Europe (Figure 7). Node 18 exhibited niche overlap outside of the regression’s 95% confidence intervals. In addition, the niche overlap values at internal nodes are significantly negatively correlated with BAMM rates of climatic niche evolution for the annual mean temperature (Bio1) (*R*^2^ = 0.790, *F*_1,6_ = 22.800, *p* < 0.01), annual mean diurnal range (Bio2) (*R*^2^ = 0.818, *F*_1,6_ = 26.880, *p* < 0.01), annual temperature range (Bio7) (*R*^2^ = 0.780, *F*_1,6_ = 21.270, *p* < 0.01), mean temperature of the wettest quarter (Bio8) (*R*^2^ = 0.792, *F*_1,6_ = 22.900, *p* < 0.01), and precipitation of the coldest quarter (Bio19) (*R*^2^ = 0.787, *F*_1,6_ = 22.190, *p* < 0.01). This indicates that niche divergence among species within a subclade is generally related to a higher rate of climatic niche evolution in the respective subclade (Figure 7b–f).

## 4. Discussion

### 4.1. Diversification and Phylogeny

The pattern of falcon species diversification through time in Europe unfolded by this work indicated an initial early-burst stage, followed by a reduction in speciation towards the more recent period, without this model deviating significantly from the expectation under a constant-rate process. The BAMM approach could not identify any shifts in falcon diversification rates in Europe and instead emphasized a reduction in estimates of instantaneous net falcon diversification rate over time, especially determined by a strong reduction in speciation rates through time within the falcon clade. The extant falcon diversity is estimated to have arisen within the last 5–7 million years, with most diversity within subgroups appearing more recently [19]. The present study indicates a rather different chronology of falcon diversity accumulation in Europe, with only two diversification points in the last two million years, during the Pleistocene (Figure 1a), and not four, as Fuchs’ phylogeny indicates [18]. These inconsistencies derive from the different methodologies used to obtain the phylogenetic trees and, implicitly, the divergence times used by the two authors [19,41], knowing that Jetz et al.’s phylogeny [41], from which we obtained our tree, suffers from several drawbacks as it does not include sequence data for some species, whose positions in the tree were randomly simulated [74]. However, by using Jetz et al.’s phylogeny [41], we tried to account for the effect of phylogenetic uncertainty, providing more reliable parameter estimates and realistic intervals around them. Moreover, assessing the evolutionary history of regional biotas (as in the present case) could raise the issue of the impact of incomplete taxon sampling on divergence time estimations [75], although incomplete taxon sampling is not a problem for phylogenetic inference [76]. Nonetheless, the divergence times between falcon species estimated by this study is in agreement with the OneZoom tree of life explorer [77].

On the other hand, the Pleistocene was relatively insignificant in terms of avian species formation in general, the emergence of modern Palearctic species (based on the presence of present-day species in the European fossil record) being positioned during the Pliocene and Early Pleistocene and very few after this period [54]. It appears that speciation and extinction rates did not increase significantly in the face of the extreme environmental instability of the Pleistocene, with diversification rates decreasing through the Pleistocene compared with the Pliocene [78]. However, this does not exclude the role of Pleistocene glacial cycles in speciation, both in initiating phylogeographic separation within species and in completing speciation that had been started earlier [79].

### 4.2. Niche Evolution

Falcons are species of warm climates, being the climate in which they presumably originated in the Neotropics, but they are also well represented in temperate and cool dry climates [54]. Actual niche positions of falcons in Europe, revealed by phylogenetic PCA analysis, showed a relative separation of species in climatic space (Figure 1d), supported by the reduction in species niche overlap, both in geographic and environmental spaces, with 56.6% of the niche overlap pairwise comparisons exhibiting significantly niche divergence in the environmental PCA space, based on a niche equivalency test. However, it also suggests a possible convergent evolution, with some species clustered together in the climatic niche space, although it is known that convergent evolution can occur for reasons unrelated to adaptation [80]. The results of this study revealed a combination of lack of phylogenetic signal for all considered climate axes (Supplementary Table S7), convergent climatic evolution, and niche divergence in the history of falcons in Europe.

#### 4.2.1. Niche Conservatism

There is no significant climatic evolutionary specialization of closely related European falcon species to a particular climatic regime in Europe based on our methodological approaches. On the other hand, PCA niche equivalency and similarity tests suggested the presence of a significant niche conservatism for *F. subbuteo*/*eleonorae* and *F. tinnunculus*/*naumanni*, and ancestral state reconstruction analysis suggested a niche conservatism for *F. cherrug*/*rusticolus* regarding precipitation seasonality (Bio15) and precipitation of the warmest quarter (Bio18). It is well known that there is some degree of conservatism in the fundamental niche of a species [9,33,81,82] in some circumstances (strong stabilizing selection; lack of genetic variation; genetic or functional constraints; lack of gene flow; pleiotropy; competition, predation and other biotic factors) [83,84,85], although a series of analyses showed that traits affecting physiological tolerances (which contribute to setting the fundamental niche) exhibit more variation in the extent of their conservatism [82,86,87]. Despite this, the current climatic distribution of *F. subbuteo*/*eleonorae* and *F. tinnunculus*/*naumanni* revealed by phylogenetic PCA analysis (Figure 1d) highlighted the importance of precipitation seasonality (Bio15), annual mean temperature (Bio1), annual mean diurnal range (Bio2), and precipitation of the warmest quarter (Bio18) as drivers in the climatic differentiation of these two closest relative species pairs in Europe (climatic variables with the highest loadings on the PC1 axis, Supplementary Table S6). These climatic divergences emerge also from the reconstruction of ancestral states in both the g-space and the e-space (Figure 2, Figure 3 and Figure 4), especially for annual mean temperature (Bio1), annual mean diurnal range (Bio2), precipitation seasonality (Bio15), and precipitation of the warmest quarter (Bio18), for both species pairs.

#### 4.2.2. Convergent Evolution

Convergent evolution for the falcon clade in Europe, as phylogenetic PCA (Figure 1d) and ancestral climatic niche reconstruction analyses revealed (Figure 2, Figure 3 and Figure 4), suggested an Ornstein–Uhlenbeck constrained model of climatic evolution. Convergent evolution is among the most powerful lines of evidence for the power of natural selection in shaping organisms to their environment [88], which is the antithesis of phylogenetic signal [81]. A single model of trait evolution cannot explain by itself the climatic evolution of falcons for the entire dataset, with OU1 and BM1 models being similarly informative (Table 2). In addition, the OU1 model is frequently incorrectly favored over simpler models when using likelihood ratio tests, mainly with datasets that are small, as in the present case [89]. Although the OU multipeak model (OUM) was a more biologically realistic model for many datasets [89], it was not statistically favored for our data, albeit climatic niche reconstruction (Figure 2, Figure 3 and Figure 4) and PGLS analyses (Figure 5) pointed out those species with subtropical and mid-latitude distributions (*F. eleonorae*, *F. naumanni*, *F. biarmicus* and, to a lesser extent, *F. cherrug*) as having the highest climatic optima, compared with those species with arctic/boreal and multilatitude distributions in Europe. This is especially true for annual mean temperature (Bio1), precipitation seasonality (Bio15), and annual mean diurnal range (Bio2), with lowest values for precipitation of the warmest quarter (Bio18) (except for *F. cherrug*), in line with their geographic distribution, and the highest degree of specialization, in accordance with the climate variability hypothesis that predicts that tropical (subtropical) organisms should have narrower physiological thermal breadths compared to organisms in temperate zones [90]. An example of convergent evolution revealed by this work was also highlighted among *F. rusticolus* and *F. columbarius*, especially for annual mean temperature (Bio1), based on ancestral niche reconstruction plots (Figure 2, Figure 3 and Figure 4) and a convergent strength test. Both species are among the falcon species most tolerant to low temperatures, their habitats stretching from a circumpolar distribution in arctic and subarctic regions (*F. rusticolus*) to low arctic and cold temperate regions, including boreal forests (*F. columbarius*).

#### 4.2.3. Niche Divergence

According to our results, the climatic niche evolution of falcons in Europe was mainly driven by niche divergence, especially for closest relative species, with BAMM highlighting an increase in the rates of climatic niche evolution especially for nodes 15 and 16 (hierofalcon subclade) of the MCCT (Figure 1a) for most of the climatic variables. Likewise, age range correlation analysis suggested that the speciation of falcons in Europe was influenced by their climatic niche differentiation, a pattern consistent with the accumulation of ecological divergence towards more recent times, expressed by less niche overlap. The examination of the scatter points for this correlation (Figure 7) indicated that more recent divergence points of the MCCT, like node 16 (ca. 1.271 Mya) and node 15 (ca. 1.817 Mya), involving the hierofalcons subclade exhibited lesser niche overlap (niche divergence) than older ones. In fact, the hierofalcons’ average niche overlap value in the environmental space is very low, indicating niche divergence, with significantly lower niche overlap between these three species over the expectation, based on the PCA niche equivalency test (but not for the niche similarity test) and also with a similar niche overlap behavior in the geographic space. A series of studies that combine phylogeny with the geographic range of species showed that the youngest sister species of many groups, including birds and mammals, often have completely nonoverlapping ranges [91]. Complementarily, Blomberg’s *K* values for all seven bioclimatic variables are below 1 (Supplementary Table S7), indicating that variance is distributed within subclades and suggesting climatic niche divergence of species and weak phylogenetic signal, knowing that a weak phylogenetic signal may result from divergent selection [92]. BAMM rate-through-time analysis also indicated a significant climatic disparity between falcons after ca. 4 Mya (Figure 6), especially for annual temperature range (Bio7), mean temperature of the wettest period (Bio8), and precipitation of the coldest quarter (Bio19). These climatic niche axes have the highest magnitude on the PC2 axis, contributing to the delineation of the hierofalcons in climatic space (Figure 1d; Supplementary Table S6). In fact, the increase in disparity in the climatic niche space in extant birds, with a sharp period in the last 4 million years, apparently started at the end of the Cretaceous Period–K-Pg boundary [2]. In the case of the hierofalcons, niche divergence is related to the rate of climatic niche evolution, with both node 15 and node 16 having a higher rate for thermic niche axes and precipitation of the coldest quarter (Bio19) compared with other nodes of the MCCT (Figure 7b–f), highlighting that the rate at which the climatic niche evolves could be related to the capacity of lineages to explore available space [93]. This also supports the finding that the increase in climatic disparity among extant birds overlapped an increase in evolutionary climatic rates through time, as a consequence of the colonization of new niche spaces [2]. The last four million years were marked by increasing climatic deterioration in the Late Pliocene and during the Pleistocene, leading to the separation of different hierofalcon ancestor populations. This period was long enough to differentiate *F. cherrug* as a temperate steppe species, with the highest mean climatic tolerance among hierofalcons for annual temperature range (Bio7) and mean temperature of the wettest quarter (Bio8) and the lowest mean for precipitation of the coldest quarter (Bio19) and *F. rusticolus* as a cold tundra species, with the lowest climatic tolerance of the three hierofalcon species for thermal niche axes and an intermediate tolerance position between them, regarding precipitation of the coldest quarter (Bio19). As a species belonging to warm and dry open habitats, *F. biarmicus* has the highest tolerance for precipitation of the coldest quarter (Bio19) among hierofalcons and an intermediate climatic tolerance value for the two thermal variables (Figure 2, Figure 3 and Figure 4).

#### 4.2.4. Rate of Climatic Niche Evolution and Climatic Change

Like other species worldwide, European falcon species are now under pressure of climatic change. Hierofalcons showed the fastest rates of climatic niche evolution, compared to other falcon subclades, indicating their resilience to cope with the future climatic challenges. From a climatic perspective, comparing generalist and specialist falcon species, specialist ones have a higher rate of climatic niche evolution, dismantling the main way through which species will be accommodated in the multidimensional climate space in the future.

## 5. Conclusions

This study uncovered that the lineage diversification of the genus *Falco* in Europe occurred at a relatively constant rate in time, while the climatic disparity, especially for thermal climatic niche axes, increased substantially, with a sharp burst ca. 4 Mya, indicating that the overall accumulation of disparity within European falcon subclades is relatively recent. This finding is confirmed by an increase in the rate of climatic niche evolution of species after that point. The climatic evolutionary history of European falcon species was not driven by niche conservatism but rather by a combination of niche divergence and convergent evolution. The accumulation of ecological divergence towards more recent times (indicating an allopatric speciation), expressed by lesser niche overlap between falcon species, and a significant correlation between niche overlap values and rates of climatic niche evolution at internal nodes of the falcon phylogeny, mostly for thermal variables, suggested that the speciation of falcons in Europe was influenced by their climatic niche differentiation. Moreover, the convergent evolution, induced by the extreme conditions that alternated over the last several millions of years in Europe, clustered the falcon species in the climatic niche space, with less closely related species occupying similar niches (specialization). Closely related falcon species, occupying mainly distinct climatic niche spaces by specialization or generalization, underwent rapid niche evolution, with a higher rate of climatic niche evolution for specialist species, giving them a potential advantage facing climate change.

## Figures and Tables

**Table 1:** Alternative evolutionary models fitted to the diversification history of the European falcon clade. Included fitted models are Yule (pure-birth model), crBD (constant-rate birth–death model), vS (variable speciation), vE (variable extinction), vSE (variable speciation and extinction), DDL + E (density-dependent linear model), and DDX + E (density-dependent exponential model). The best-fit model selections are based on ΔAICc scores. *λ*: speciation rate; *μ*: extinction rate. For vS, vE, and vSE models, the values in parentheses represent the estimated values of λ and μ at the root of the tree.

Model	logL	*λ*	*μ*	AICc	ΔAICc
Yule	−13.329	0.103	0	28.658	0
crBD	−13.330	0.105	<0.001	30.660	2.002
vS	−12.575	−0.167 (0.108)	0.339	31.151	2.493
vE	−13.329	0.103	0.081 (<0.001)	32.658	4.000
vSE	−12.549	−0.209 (0.100)	−0.075 (0.295)	33.098	4.440
DDL + E	−25.114	0.262	0	54.228	25.570
DDX + E	−24.365	10.248	0.070	54.731	26.073

**Table 2:** Comparison of five evolutionary model fits for climatic variables. Models were fitted over 1000 stochastically mapped trees (obtained by running 10 stochastic character histories on 100 randomly selected trees from the posterior distribution of the fully resolved MCMC search) with a single topology. For all models, loglik (maximum log-likelihood), AIC_c_ (Akaike information criterion corrected for sample sizes), and ΔAIC_c_ (AIC_c_ score of the best-fit model minus the AIC_c_ score of the remaining models) represent values averaged over 1000 simulations. The supports for each model within a given set of models for each variable are indicated by AIC_c_ weight, ranging from 0 (no support) to 1 (full support). For each variable, the best-fit model based on the lowest AIC_c_ score is marked by bold text. See text for variable descriptions.

Trait	Model	Loglik	AIC_c_	ΔAIC_c_	AIC_c_ Weight
Bio1	BM1 ^e^	−28.402 ± 1.407	62.520 ± 2.814	1 ± 2.681	0.352
	BMM **	−27.355 ± 3.495	70.711 ± 6.990	9.191 ± 6.857	0.004
	**OU1**	**−25.760 ± 0.067**	**61.520 ± 0.133**	**0 ± 0**	**0.617**
	OUM **	−22.549 ± 2.670	70.098 ± 5.341	8.578 ± 5.208	0.011
	EB **	−29.265 ± 1.978	68.531 ± 3.957	7.011 ± 3.824	0.016
Bio2	BM1 ^e^	−14.232 ± 1.761	34.178 ± 0.112	0.744 ± 0.183	0.445
	BMM **	−12.990 ± 3.532	41.980 ± 7.065	8.546 ± 6.76	0.008
	**OU1**	**−11. 717 ± 0.152**	**33.434 ± 0.305**	**0 ± 0**	**0.519**
	OUM **	−8.554 ± 2.573	42.109 ± 5.146	8.675 ± 4.841	0.007
	EB *	−15.123 ± 1.841	40.246 ± 3.683	6.812 ± 3.378	0.021
Bio7	BM1 *	−18.660 ± 2.247	43.035 ± 4.495	3.286 ± 4.237	0.159
	BMM ***	−16.958 ± 4.152	49.917 ± 8.305	10.168 ± 8.047	0.006
	**OU1**	**−14.874 ± 0.129**	**39.749 ± 0.258**	**0 ± 0**	**0.825**
	OUM ***	−13.198 ± 1.781	51.396 ± 3.562	11.647 ± 3.304	0.002
	EB **	−19.634 ± 2.572	49.269 ± 5.144	9.52 ± 4.886	0.008
Bio8	BM1 *	−27.566 ± 2.431	60.846 ± 4.862	4.43 ± 4.734	0.098
	BMM ***	−25.993 ± 3.755	67.987 ± 7.511	11.571 ± 7.383	0.002
	**OU1**	**−23.208 ± 0.064**	**56.416 ± 0.128**	**0 ± 0**	**0.893**
	OUM ***	−21.406 ± 1.672	67.813 ± 3.344	11.397 ± 3.216	0.003
	EB ***	−28.670 ± 2.624	67.340 ± 5.248	10.924 ± 5.12	0.004
Bio15	BM1 ^e^	−32.143 ± 1.767	70.001 ± 3.543	1.796 ± 3.324	0.277
	BMM **	−30.654 ± 3.086	77.308 ± 6.172	9.103 ± 5.953	0.007
	**OU1**	**−29.102 ± 0.109**	**68.205 ± 0.219**	**0 ± 0**	**0.679**
	OUM **	−26.022 ± 2.424	77.044 ± 4.848	8.839 ± 4.629	0.008
	EB *	−32.221 ± 1.305	74.443 ± 2.611	6.238 ± 2.392	0.029
Bio18	BM1 *	−55.786 ± 1.498	116.062 ± 2.99	2.345 ± 2.778	0.227
	BMM **	−53.178 ± 4.174	122.357 ± 8.349	8.64 ± 8.137	0.009
	**OU1**	**−51.858 ± 0.106**	**113.717 ± 0.212**	**0 ± 0**	**0.731**
	OUM **	−48.924 ± 2.577	122.848 ± 5.155	9.131 ± 4.943	0.007
	EB *	−55.173 ± 1.498	120.347 ± 2.997	6.63 ± 2.785	0.026
Bio19	BM1 *	−51.445 ± 1.990	108.605 ± 3.981	5.604 ± 3.895	0.058
	BMM ***	−48.910 ± 4.396	113.821 ± 8.793	10.82 ± 8.707	0.004
	**OU1**	**−46.500 ± 0.043**	**103.001 ± 0.086**	**0 ± 0**	**0.929**
	OUM ***	−44.660 ± 1.846	114.320 ± 3.692	11.319 ± 3.606	0.003
	EB **	−51.445 ± 1.990	112.891 ± 3.981	9.89 ± 3.895	0.006

Notes: The differences between the best-fit model and the rest of the models for each climatic trait were abbreviated as follows (based on Burnham and Anderson [64]): ^e^ equivalent models. * more or less distinguishable models. ** distinguishable models. *** different models.

## Data Availability

The climatic data used in this study were downloaded from the WorldClim database (https://www.worldclim.org, accessed on 2 January 2022). Species data were obtained from the Global Biodiversity Information Facility—GBIF (https://www.gbif.org, accessed on 1 April 2022) and phylogenetic information was obtained from BirdTree.org (https://birdtree.org, accessed on 12 March 2022).

## Supplementary Materials

Supplementary Materials: The following supporting information can be downloaded at: https://www.mdpi.com/article/10.3390/biology13020113/s1. Table S1: List of WorldClim’s bioclimatic variables used in this study. In bold, variables retained for further analyses after controlling for collinearity; Table S2: Median climatic values extracted from the species occurrence points, for each climatic variable; Table S3: Weighted means of the predicted niche occupancy (PNO) for all falcon species and for all bioclimatic variable included in our analyses; Table S4: Niche overlap among falcon species in geographic space based on Schoener’s D metric; Table S5: Niche overlap: niche equivalency and similarity test for falcon clade, according to Broennimann et al. (2012) [34] approach; Table S6: Loadings of phylo-genetic principal component analysis of climatic axes; Table S7: Results of tests of phylogenetic signal for bioclimatic variables; Figure S1: Predicted falcon species distribution in Europe based on Bioclim algorithm; Figure S2: The relationship between range size and niche breadth for falcon species using Phylogenetic Generalized Least Squares (PGLS); Figure S3: BAMM phylorate plots showing rates of climatic niche evolution for genus Falco in Europe.
