# Supplementary material for: Exploring the Climatic Niche Evolution of the Genus Falco (Aves: Falconidae) in Europe
Source: Biology (Basel). 2024 Feb 11;13(2):113. doi: 10.3390/biology13020113 (PMC10886973; doi:10.3390/biology13020113)
Supplement: Supplementary file 1 [file biology-13-00113-s001.zip › biology-2850248-supplementary.pdf]

Supplementary Material

Exploring the climatic niche evolution of the genus *Falco* (Aves: Falconidae) in Europe

Simona Mariana Popescu, Cristian Tigae, Aurelian Dobrițescu, Dragoș Mihail Ștefănescu *

*** Correspondence:** dragos.stefanescu@edu.ucv.ro

Supplementary Figures and Tables

Table S1. List of WorldClim' bioclimatic variables used in this study. In bold, variables retained for further analyses after controlling for collinearity

Variable	Abbrev.
**Annual mean temperature (ºC)**	Bio 1
**Annual mean diurnal range (mean of the monthly temperature ranges (max temp – min temp)) (ºC)**	Bio 2
Isothermality ((Bio 2/Bio 7) * 100) (%)	Bio 3
Temperature seasonality (standard deviation * 100) (ºC)	Bio 4
Maximum temperature of warmest month (ºC)	Bio 5
Minimum temperature of coldest month (ºC)	Bio 6
**Temperature annual range (Bio 5 – Bio 6) (ºC)**	Bio 7
**Mean temperature of wettest quarter (ºC)**	Bio 8
Mean temperature of driest quarter (ºC)	Bio 9
Mean temperature of warmest quarter (ºC)	Bio 10
Mean temperature of coldest quarter (ºC)	Bio 11
Annual precipitation (mm)	Bio 12
Precipitation of wettest month (mm)	Bio 13
Precipitation of driest month (mm)	Bio 14
**Precipitation seasonality (standard deviation * 100)**	Bio 15
Precipitation of wettest quarter (mm)	Bio 16
Precipitation of driest quarter (mm)	Bio 17
**Precipitation of warmest quarter (mm)**	Bio 18
**Precipitation of coldest quarter (mm)**	Bio 19

Table S2. Median climatic values extracted from the species occurrence points, for each climatic variable

Species	Variable						
	Bio1	Bio2	Bio7	Bio8	Bio15	Bio18	Bio19
F. biarmicus	13.591	8.665	25.882	11.090	31.197	95.000	188.000
F. cherrug	9.469	9.492	30.826	18.289	26.568	190.000	112.000
F. columbarius	2.474	7.698	30.911	10.790	30.078	218.500	131.000
F. eleonorae	14.753	9.195	25.183	12.581	36.000	95.000	178.000
F. naumanni	14.727	10.896	28.391	10.239	48.940	55.000	207.000
F. peregrinus	10.741	9.132	26.429	10.420	29.862	163.000	194.000
F. rusticolus	1.200	6.658	22.419	7.206	25.048	206.500	168.500
F. subbuteo	9.365	8.617	27.657	13.027	27.899	193.000	151.000
F. tinnunculus	9.552	8.737	27.380	11.864	28.893	188.000	164.000
F. vespertinus	10.927	9.765	30.457	16.229	29.713	158.500	121.000
Mean climatic tolerance	9.679	8.885	27.553	12.173	31.419	156.250	161.450

Table S3. Weighted means of the predicted niche occupancy (PNO) for all falcon species and for all bioclimatic variable included in our analyses

Species	Variable						
	Bio 1	Bio 2	Bio 7	Bio 8	Bio 15	Bio 18	Bio 19
F. biarmicus	11.868	9.328	26.981	10.384	35.768	124.127	201.675
F. cherrug	8.471	9.121	30.904	17.493	30.017	206.460	114.452
F. columbarius	3.787	7.676	28.729	10.247	28.694	222.621	167.221
F. eleonorae	12.215	9.436	27.264	12.112	36.235	123.136	187.170
F. naumanni	13.141	10.291	28.010	10.224	42.341	86.078	201.310
F. peregrinus	9.023	8.875	27.647	11.836	30.176	181.576	175.501
F. rusticolus	2.847	7.486	27.797	8.394	27.702	216.300	174.833
F. subbuteo	8.345	8.745	28.372	13.520	28.110	196.811	152.977
F. tinnunculus	8.488	8.787	28.246	12.847	29.285	189.806	159.942
F. vespertinus	9.679	9.311	29.324	14.477	31.527	173.503	144.747
Mean climatic tolerance	8.787	8.906	28.327	12.153	31.985	172.041	167.982

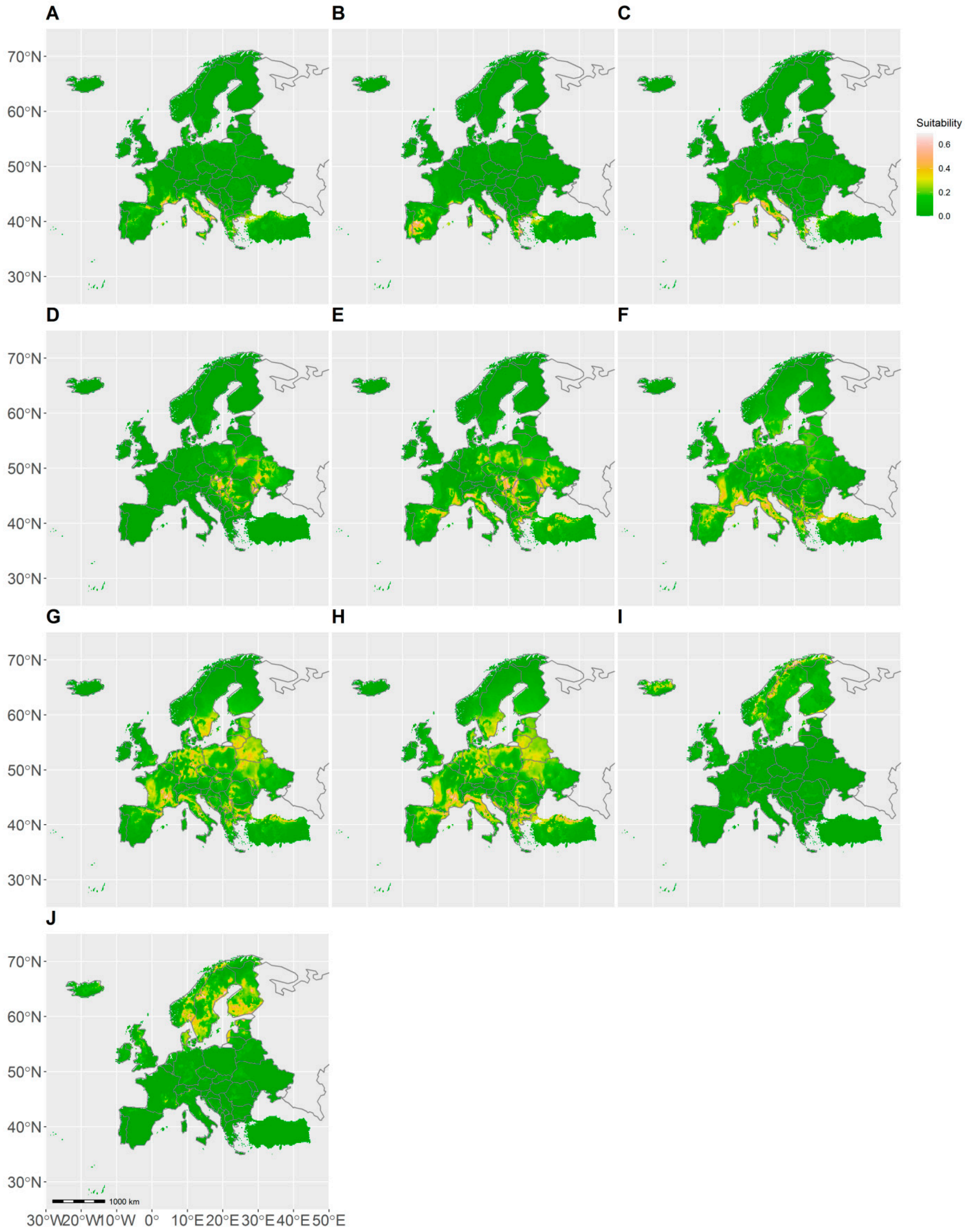

Figure S1. Predicted falcon species distribution in Europe based on Bioclim algorithm: *Falco biarmicus* (A); *Falco naumanni* (B); *Falco eleonorae* (C); *Falco cherrug* (D); *Falco vespertinus* (E); *Falco peregrinus* (F); *Falco subbuteo* (G); *Falco tinnunculus* (H); *Falco rusticolus* (I), and *Falco columbarius* (J). According to legend, warmer colours indicate a higher probability of species occurrence.

Table S4. Niche overlap among falcon species in geographic space based on Schoener`s *D* metric

s	*F. columbarius*	*F. tinnunculus*	*F. naumanni*	*F. vespertinus*	*F. subbuteo*	*F. eleonorae*	*F. peregrinus*	*F. biarmicus*	*F. cherrug*	*F. rusticolus*
F. columbarius		0.296	0.050	0.133	0.266	0.053	0.297	0.082	0.080	0.682
F. tinnunculus			0.316	0.631	0.881	0.442	0.841	0.371	0.352	0.221
F. naumanni				0.354	0.260	0.593	0.390	0.552	0.067	0.040
F. vespertinus					0.603	0.511	0.594	0.399	0.523	0.088
F. subbuteo						0.396	0.739	0.317	0.389	0.188
F. eleonorae							0.479	0.623	0.187	0.053
F. peregrinus								0.456	0.277	0.226
F. biarmicus									0.098	0.058
F. cherrug										0.030
F. rusticolus										

Table S5. Niche overlap, niche equivalency and similarity test for falcon clade, according to Broennimann et al. (2012) approach. Lower values of *D* (equivalency/similarity test, with significant values in bold when $p < 0.05$) indicating niche divergence (the niche overlap is less equivalent/similar than random), and greater value (equivalency/similarity test, with significant values in bold when $p < 0.05$) indicating niche conservatism (the niche overlap is more equivalent/similar than random

**Species 1**	**Species 2**	**Niche overlap (*D*)**	**Niche equivalency test**		**Niche similarity test**			
			**Niche conservatism (greater)**	**Niche divergence (lower)**	**Niche conservatism (greater)**		**Niche divergence (lower)**	
			**p-value for *D***	**p-value for *D***	**p-value for *D* (1-2)**	**p-value for *D* (2-1)**	**p-value for *D* (1-2)**	**p-value for *D* (2-1)**
F.columbarius	*F.biarmicus*	0.060	1.000	0.009	0.591	0.576	0.407	0.4025
	F.cherrug	0.137	1.000	0.009	0.542	0.311	0.441	0.683
	F.eleonorae	0.041	1.000	0.009	0.329	0.528	0.719	0.469
	F.naumanni	0.040	1.000	0.009	0.908	0.332	0.071	0.678
	F.peregrinus	0.245	1.000	0.009	0.338	0.002	0.662	1.000
	F.rusticolus	0.414	0.990	0.019	0.022	0.068	0.980	0.926
	F.subbuteo	0.287	0.980	0.029	0.316	0.119	0.713	0.896
	F.tinnunculus	0.298	0.534	0.465	0.297	0.018	0.709	0.985

	F.vespertinus	0.179	0.950	0.108	0.394	0.135	0.590	0.872
F.biarmicus	*F.cherrug*	0.065	1.000	0.009	0.439	0.493	0.529	0.519
	F.eleonorae	0.683	0.198	0.821	0.005	0.014	0.993	0.990
	F.naumanni	0.556	0.207	0.742	0.042	0.105	0.965	0.893
	F.peregrinus	0.382	0.415	0.504	0.020	0.035	0.980	0.969
	F.rusticolus	0.111	1.000	0.009	0.303	0.440	0.694	0.569
	F.subbuteo	0.406	0.049	0.980	0.050	0.043	0.950	0.958
	F.tinnunculus	0.358	0.396	0.554	0.037	0.019	0.972	0.986
	F.vespertinus	0.527	0.019	1.000	0.140	0.091	0.860	0.912
F.cherrug	*F.eleonorae*	0.155	1.000	0.019	0.187	0.212	0.820	0.803
	F.naumanni	0.025	1.000	0.009	0.661	0.316	0.323	0.632
	F.peregrinus	0.031	1.000	0.009	0.262	0.434	0.732	0.577
	F.rusticolus	0.095	1.000	0.009	0.693	0.694	0.299	0.324
	F.subbuteo	0.090	1.000	0.009	0.094	0.319	0.889	0.706
	F.tinnunculus	0.060	1.000	0.009	0.062	0.289	0.947	0.724

	F.vespertinus	0.156	1.000	0.009	0.005	0.248	0.994	0.749
F.eleonorae	*F.naumanni*	0.448	1.000	0.019	0.116	0.480	0.876	0.580
	F.peregrinus	0.364	0.712	0.267	0.029	0.018	0.965	0.976
	F.rusticolus	0.086	1.000	0.009	0.509	0.278	0.483	0.768
	F.subbuteo	0.395	0.009	1.000	0.099	0.006	0.907	0.996
	F.tinnunculus	0.326	0.445	0.504	0.024	0.004	0.976	0.999
	F.vespertinus	0.492	0.049	0.950	0.176	0.061	0.839	0.926
F.naumanni	*F.peregrinus*	0.448	0.089	0.940	0.091	0.079	0.916	0.917
	F.rusticolus	0.026	1.000	0.009	0.487	0.893	0.528	0.112
	F.subbuteo	0.392	0.009	1.000	0.125	0.191	0.867	0.807
	F.tinnunculus	0.428	0.009	1.000	0.023	0.057	0.984	0.935
	F.vespertinus	0.540	0.009	1.000	0.039	0.047	0.963	0.959
F.peregrinus	*F.rusticolus*	0.108	1.000	0.009	0.170	0.278	0.847	0.703
	F.subbuteo	0.719	1.000	0.009	0.008	0.013	0.992	0.990
	F.tinnunculus	0.846	0.554	0.306	0.001	0.004	1.000	1.000

	F.vespertinus	0.486	1.000	0.009	0.182	0.063	0.817	0.912
F.rusticolus	*F.subbuteo*	0.263	0.821	0.168	0.266	0.031	0.770	0.958
	F.tinnunculus	0.164	1.000	0.009	0.332	0.088	0.693	0.924
	F.vespertinus	0.113	0.980	0.019	0.475	0.249	0.494	0.740
F.subbuteo	*F.tinnunculus*	0.732	1.000	0.009	0.017	0.008	0.991	0.997
	F.vespertinus	0.548	0.217	0.762	0.208	0.073	0.768	0.922
F.tinnunculus	*F.vespertinus*	0.524	0.178	0.881	0.084	0.049	0.940	0.966

Table S6. Loadings of phylogenetic principal components analysis of climatic axes

Variable	PC1	PC2
annual mean temperature (Bio 1)	-0.845	-0.504
annual mean diurnal range (Bio 2)	-0.719	-0.687
annual temperature range (Bio 7)	0.426	-0.885
mean temperature of wettest quarter (Bio 8)	0.132	-0.986
precipitation seasonality (Bio 15)	-0.965	-0.101
precipitation of warmest quarter (Bio 18)	0.997	-0.040
precipitation of coldest quarter (Bio 19)	-0.578	0.814

Table S7. Results of tests of phylogenetic signal for bioclimatic variables

Bioclimatic variable	Pagel's λ		Blomberg's *K*		Moran's *I*		Abouheif's C_{mean}	
	λ	*p*- value	*K*	*p*- value	*I*	*p*- value	C_{mean}	*p*- value
Bio1	7.31605e-05	1	0.280	0.727	-0.181	0.590	0.016	0.440
Bio2	7.31605e-05	1	0.300	0.747	-0.231	0.754	-0.113	0.513
Bio7	7.31605e-05	1	0.166	0.966	-0.294	0.887	-0.252	0.931
Bio8	7.31605e-05	1	0.149	0.992	-0.369	0.943	-0.303	0.969
Bio15	7.31605e-05	1	0.392	0.550	-0.321	0.921	-0.170	0.828
Bio18	7.31605e-05	1	0.356	0.585	-0.255	0.723	-0.082	0.619
Bio19	7.31605e-05	1	0.193	0.947	-0.369	0.945	-0.303	0.958

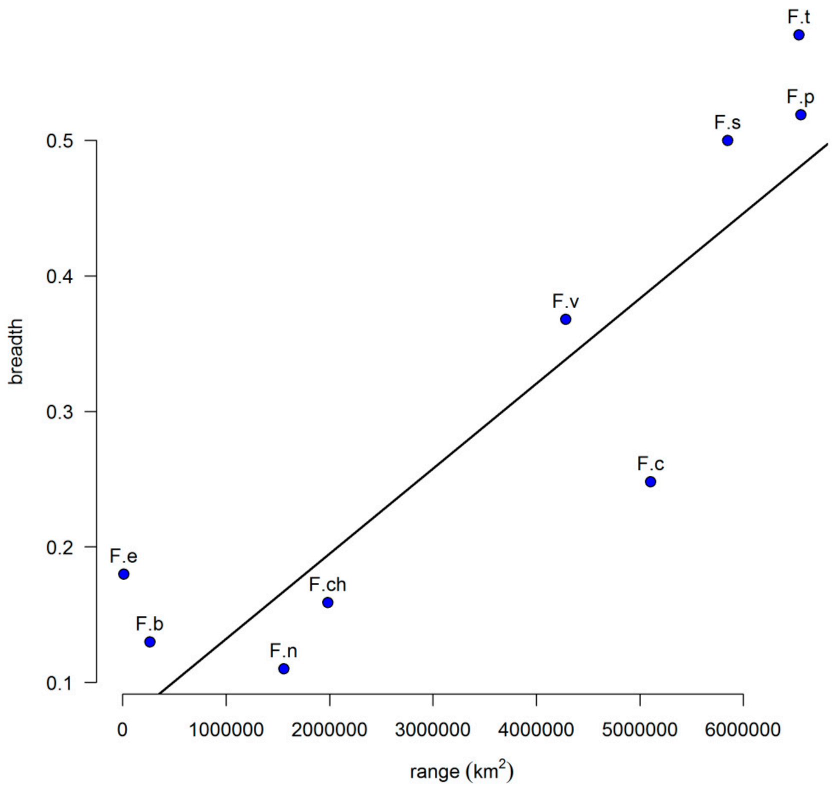

Figure S2. The relationship between range size and niche breadth for falcon species using Phylogenetic Generalized Least Squares (PGLS). Species names are indicated by first letter of the genus and the first letter of the species name, except for *F. cherrug*, in which case the name of the species is indicated with two letters (*F. ch*), in order to distinguish it from *F. columbarius* (*F.c*)

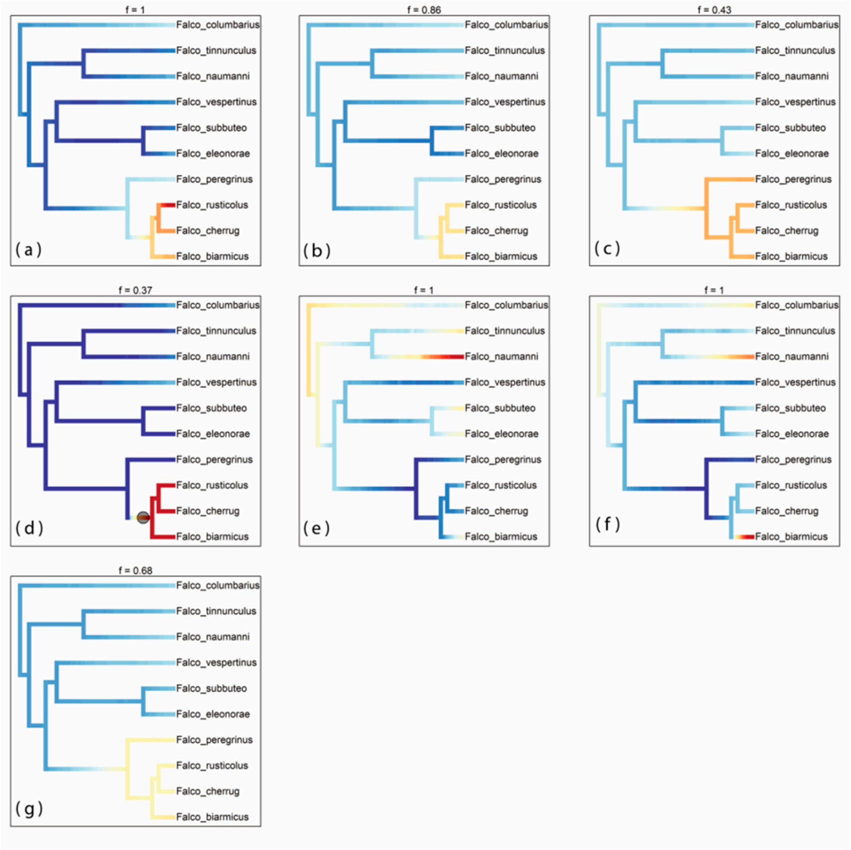

Figure S3. BAMM phylorate plots showing rates of climatic niche evolution for genus *Falco* in Europe: (a) annual mean temperature, (b) annual mean diurnal range, (c) annual temperature range, (d) mean temperature of wettest quarter, (e) precipitation seasonality, (f) precipitation of warmest quarter, and (g) precipitation of coldest quarter. Branch colours indicate instantaneous rates, with warmer colour for faster rates. The grey circle depict the rate shift point of climatic evolution detected by BAMM analysis. For each climatic variable is plotted the distinct shift configuration with the highest posterior probability (*f*, indicated above each panel).
